# Plasma proteomics reveals gestational age-specific responses to mechanical ventilation and identifies the mechanistic pathways that initiate preterm lung injury

**DOI:** 10.1038/s41598-018-30868-x

**Published:** 2018-08-22

**Authors:** Prue M. Pereira-Fantini, Sean G. Byars, Karen E. McCall, Elizabeth J. Perkins, Regina B. Oakley, R. L. Dellacà, Peter A. Dargaville, Peter G. Davis, Vera Ignjatovic, David G. Tingay

**Affiliations:** 1 Neonatal Research, Murdoch Childrens Research Institute, Parkville, Australia; 2 Department of Paediatrics, University of Melbourne, Parkville, Australia; 3 Department of Pathology, University of Melbourne, Parkville, Australia; 4 Centre for Systems Genomics, University of Melbourne, Parkville, Australia; 5 University College Dublin, Dublin, Ireland; 6 Laboratorio di Tecnologie Biomediche, Dipartimento di Elettronica, Informazione e Ingegneria Biomedica-DEIB, Politecnico di Milano University, Milano, Italy; 7 Menzies Institute for Medical Research, University of Tasmania, Hobart, Australia; 8 The Royal Women’s Hospital, Parkville, Australia; 9 Department of Obstetrics and Gynaecology, University of Melbourne, Parkville, Australia; 10 Haematology Research, Murdoch Childrens Research Institute, Parkville, Australia; 11 Department of Neonatology, Royal Children’s Hospital, Parkville, Australia

## Abstract

The preterm lung is particularly vulnerable to ventilator-induced lung injury (VILI) as a result of mechanical ventilation. However the developmental and pathological cellular mechanisms influencing the changing patterns of VILI have not been comprehensively delineated, preventing the advancement of targeted lung protective therapies. This study aimed to use SWATH-MS to comprehensively map the plasma proteome alterations associated with the initiation of VILI following 60 minutes of standardized mechanical ventilation from birth in three distinctly different developmental lung states; the extremely preterm, preterm and term lung using the ventilated lamb model. Across these gestations, 34 proteins were differentially altered in matched plasma samples taken at birth and 60 minutes. Multivariate analysis of the plasma proteomes confirmed a gestation-specific response to mechanical ventilation with 79% of differentially-expressed proteins altered in a single gestation group only. Six cellular and molecular functions and two physiological functions were uniquely enriched in either the extremely preterm or preterm group. Correlation analysis supported gestation-specific protein-function associations within each group. In identifying the gestation-specific proteome and functional responses to ventilation we provide the founding evidence required for the potential development of individualized respiratory support approaches tailored to both the developmental and pathological state of the lung.

## Introduction

With advances in perinatal care, preterm birth has now become a major public health issue^1^. Fundamentally, the multi-organ problems of prematurity relate to exposing developmentally immature systems to ex-uterine life. Of these, the respiratory system is the most acutely compromised in early life, being structurally immature, surfactant deficient and prone to collapse^2^. These defects often necessitate the application of mechanical respiratory support in the form of positive pressure ventilation to sustain life. However, assisted ventilation is an unnatural process, and when applied to the fragile, under-developed alveolar structure of the preterm lung, causes ventilator-induced lung injury (VILI) and subsequent bronchopulmonary dysplasia (BPD)^3,4^. Even a single large lung inflation can initiate VILI in the preterm lung^5^. The consequences of VILI development in the immediate postnatal period include inhibition of surfactant function^6,7^, impaired angiogenesis^8^ and lung growth^9^ and increased oxygen need^10^, thereby committing the infant to continuing respiratory support which perpetuates lung injury. In the long-term, VILI-related lung abnormalities manifest as chronic lung disease in infancy^11^, with reduced pulmonary function^12,13^ into adult life^14^.

Minimizing VILI remains a key goal of neonatal intensive care. Whilst major advances, such as exogenous surfactant^15^, antenatal steroids^16^, better nutritional support^17^ and non-invasive ventilation^18^, have significantly improved outcomes, more than 50% of extremely preterm infants (<28 weeks gestational age (GA)) still develop BPD^19^. VILI is a multi-factorial process involving a complex interplay between multiple pathophysiological and patient-specific factors. In the preterm infant, VILI pathogenesis is specifically linked to immaturity of lung tissue, direct mechanical injury, oxidant injury, and proinflammatory mediators^15^. Therefore, identification of VILI-associated mediators and pathways will be paramount in improving respiratory outcomes for those born prematurely. Proteomics, with its ability to elucidate pathological pathways and determine protein alterations associated with specific development and/or disease states, is perfectly suited to uncovering the intermediaries associated with VILI development within the premature lung.

Plasma is increasingly used in proteomic studies of lung disease as it is easily accessible and contains measureable tissue-derived proteins with the potential to delineate disease-associated mechanisms and pathophysiology^20^. Proteomic studies of plasma using traditional label-dependent methods have been challenging, with labels themselves often co-selected^21^ and difficulties in identifying and quantifying low abundance proteins from samples in which albumin, α2-macroglobulin, transferrin, and immunoglobulins, may represent as much as 80% of the total serum protein^22^. In contrast, sequential window acquisition of all theoretical mass spectra (SWATH-MS) is a label-free data acquisition method which generates, in a single measurement, a complete recording of the fragment ion spectra of all the analytes in a biological sample^23^ and has been shown to outperform alternative methods in plasma quantification, reducing single variation, and markedly increasing the number of precisely quantified peptides^21^.

In this study we aimed to use SWATH-MS to (1) characterize alterations in the plasma proteome associated with the application of a uniform mechanical ventilation strategy to extremely preterm, preterm or term lambs and (2) identify associations between observed proteome alterations and key functional outcomes. We present here the first comprehensive map of the ventilation-induced plasma proteome after preterm birth, and reveal a GA-specific response to mechanical ventilation. Furthermore, using integrated bionformatic analysis we have identified unique GA-specific protein-function associations.

## Results

A total of 22 lambs delivered from 15 ewes were studied. No ewes had evidence of sepsis or chorioamnionitis. Birth weight increased with gestational age. Drained lung fluid volumes and measures of fetal wellbeing were comparable between groups (Supplementary Table 1).

### GA-specific functional alterations were observed following ventilation

GA-specific alterations in gas exchange included increased oxygenation index (OI) and alveolar-arterial oxygen difference (AaDO_2_) in extremely preterm and preterm animals when compared with term animals (Table 1). Morphologically, total lung weight increased and alveolar septal thickness decreased with GA.

**Table 1:** Respiratory characteristics at study conclusion.

		Extremely preterm	Preterm	Term
Gas exchange	Fraction of inspired oxygen (FiO_2_; %)	0.39 ± 0.09^*†^	0.28 ± 0.07	0.21 ± 0.00
	Peripheral oxygen saturation/FiO_2_ (SpO_2_/FiO_2_; %)	220.0 ± 55.5^*†^	338.4 ± 81.7^‡^	459.5 ± 9.9
	Oxygen saturation (SpO_2_)	90.9 ± 1.1^†^	91.4 ± 3.4^‡^	96.5 ± 2.1
	Alveolar-arterial oxygen difference (AaDO_2_)	202.6 ± 73.6^*†^	101.3 ± 53.9^‡^	22.2 ± 13.3
	Oxygenation index (OI)	18.37 ± 5.29^*†^	12.93 ± 3.40^‡^	3.88 ± 1.14
	Minute ventilation (ml/kg/min)	432 (407, 460)	131 (89, 429)	268 (188, 455)
	Tidal volume (ml/kg)	7.23 ± 0.42	6.79 ± 0.56	6.03 ± 1.37
Lung Mechanics	Lung volume at 35 cm H_2_O (ml/kg)	35.7 ± 5.9	39.5 ± 11.5	41.0 ± 6.7
	Static compliance (ml/kg/cmH_2_O)	1.04 ± 0.16	1.14 ± 0.37	1.17 ± 0.19
Airway characteristics	% airway space	62.9 ± 3.0^*†^	72.5 ± 2.8	70.9 ± 3.3
	Alveolar sac diameter (μm)	50.1 ± 7.4	58.6 ± 10.9	48.0 ± 6.5
	Alveolar sac area (μm^2^)	3596 ± 915	4327 ± 670^‡^	2664 ± 663
	Alveolar sac area (CoV)	107 (65, 184)	76 (69, 114)	91 (83, 107)
Lung morphology	Total lung weight (g)	79.0 ± 9.6^†^	89.6 ± 4.8^‡^	137.8 ± 19.4
	% lung tissue	32.2 ± 9.1	33.0 ± 4.8	38.1 ± 2.6
	Lung tissue area (μm^2^)	65768 ± 28021^†^	88948 ± 6930	97641 ± 5738
	Alveolar septal thickness (μm)	8.34 ± 0.28^†^	6.95 ± 0.51^‡^	5.12 ± 0.57
Molecular markers of lung injury (relative expression; ΔΔCt)	CYR61 gene expression; non-dependent lung	4.72 ± 2.82	5.58 ± 2.37	6.42 ± 5.44
	CYR61 gene expression; lower lung	0.98 (0.40, 1.66)	1.70 (1.22, 3.05)	1.91 (1.05, 3.45)
	CTGF gene expression; non-dependent lung	4.85 ± 2.79	4.77 ± 2.10	6.86 ± 5.23
	CTGF gene expression; gravity-dependent lung	0.95 ± 0.59^†^	1.08 ± 0.25^‡^	2.02 ± 0.90
	EGR1 gene expression; non-dependent lung	3.79 ± 2.20	6.14 ± 4.93	2.89 ± 2.32
	EGR1 gene expression; gravity-dependent lung	1.14 (0.71, 2.56)	1.73 (1.61, 6.14)	0.96 (0.61, 1.90)
	IL1B gene expression; non-dependent lung	21.18 ± 13.99	20.92 ± 6.12	10.49 ± 8.48
	IL1B gene expression; gravity-dependent lung	8.13 ± 6.58	8.43 ± 2.77	3.24 ± 3.28
	IL6 gene expression; non-dependent lung	5.84 ± 4.10^†^	3.63 ± 2.75	1.29 ± 1.14
	IL6 gene expression; gravity-dependent lung	1.43 ± 1.46	0.93 ± 0.33	0.18 ± 0.11
	IL8 gene expression; non-dependent lung	15.26 (7.84, 30.95)	16.52 (11.68, 25.80)	5.78 (3.37, 25.44)
	IL8 gene expression; gravity-dependent lung	1.88 (0.43, 2.85)	2.70 (1.73, 3.38)	0.33 (0.14, 1.65)
Other injury markers	Number of detached epithelial cells (per 15 fields of view)	18 (12, 29)^†^	5 (5, 18)	5 (4, 10)
	BALF; total protein (μg/ml)	424.4 ± 22.3^†^	352.1 ± 108.6^‡^	130.2 ± 18.7

Parametric data are represented as means ± SD (parametric) and non-parametric data as median (interquartile range). P < 0.05 in *extremely preterm vs preterm; ^†^extremely preterm vs term; ^‡^preterm vs term groups as determined by parametric or non-parametric ANOVA as appropriate (one-way ANOVA or Kruskall-Wallis ANOVA, respectively) with post hoc testing to identify intergroup differences (Tukey’s post hoc test or Dunn’s multiple comparison test, respectively). N = 7–8/group.

The total protein concentration of bronchoalveolar lung fluid (BALF) was 3-fold higher in extremely preterm and preterm lambs when compared with term lambs, whilst the number of detchaced epithelial cells was increased 4-fold in extremely preterm lambs when compared to term lambs. Gene expression of interleukin-6 (IL6) was increased five-fold in non-dependent lung tissue from extremely preterm animals compared to term animals, conversely gene expression of connective tissue growth factor (CTGF) was decreased two-fold in gravity-dependent lung tissue from either extremely preterm or preterm animals when compared to term.

### SWATH-MS identification of the plasma proteome confirmed a GA-specific ventilation response

**Figure 1:**
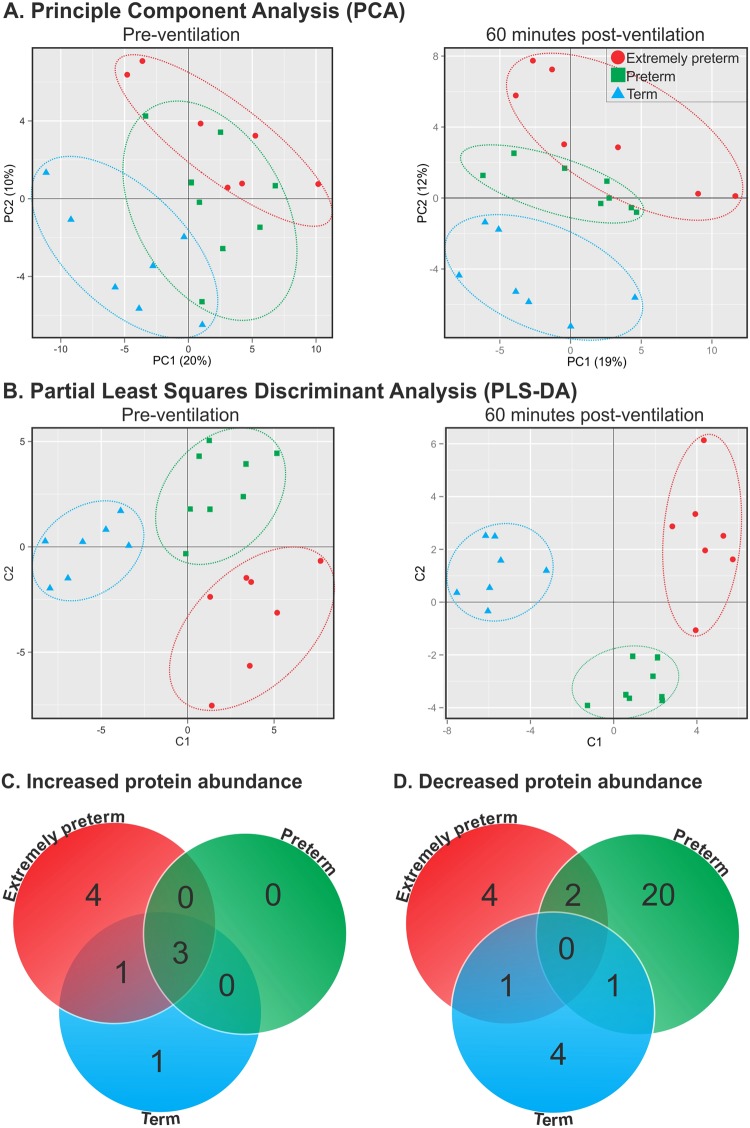
Multi-variate analysis confirmed GA-specificity of the plasma proteome response to ventilation. Multi-variate analysis of SWATH-MS data included (**A**) principle component analysis (PCA) and (**B**) partial least squares discriminant analysis (PLS-DA). Venn diagrams depicting the number of proteins expressing increased (**C**) or decreased (**D**) differential expression in response to ventilation as determined by paired t-test, p < 0.05. N = 7–8/group.

A total of 125 proteins were identified via SWATH-MS with identification confidence ≥99% and false discovery rate ≤1% (Supplementary Table 2). Unsupervised principal component analysis displayed minor overlap between the GA proteomes prior to ventilation, with GA-groups distinguishable from each other in supervised partial least squares discriminat analysis plots (Fig. 1A,B; left plot). Following ventilation, wider separation between each GA-specific proteome was observed (Fig. 1A,B; right plot).

40/125 proteins (32%) were differentially expressed in response to ventilation, including eight up-regulated (Fig. 1C) and 32 down-regulated proteins (Fig. 1D). As shown in Supplementary Table 2, 70% of proteins identified as differentially-expressed in the ventilated lamb model were also associated with human acute lung injury.

**Figure 2:**
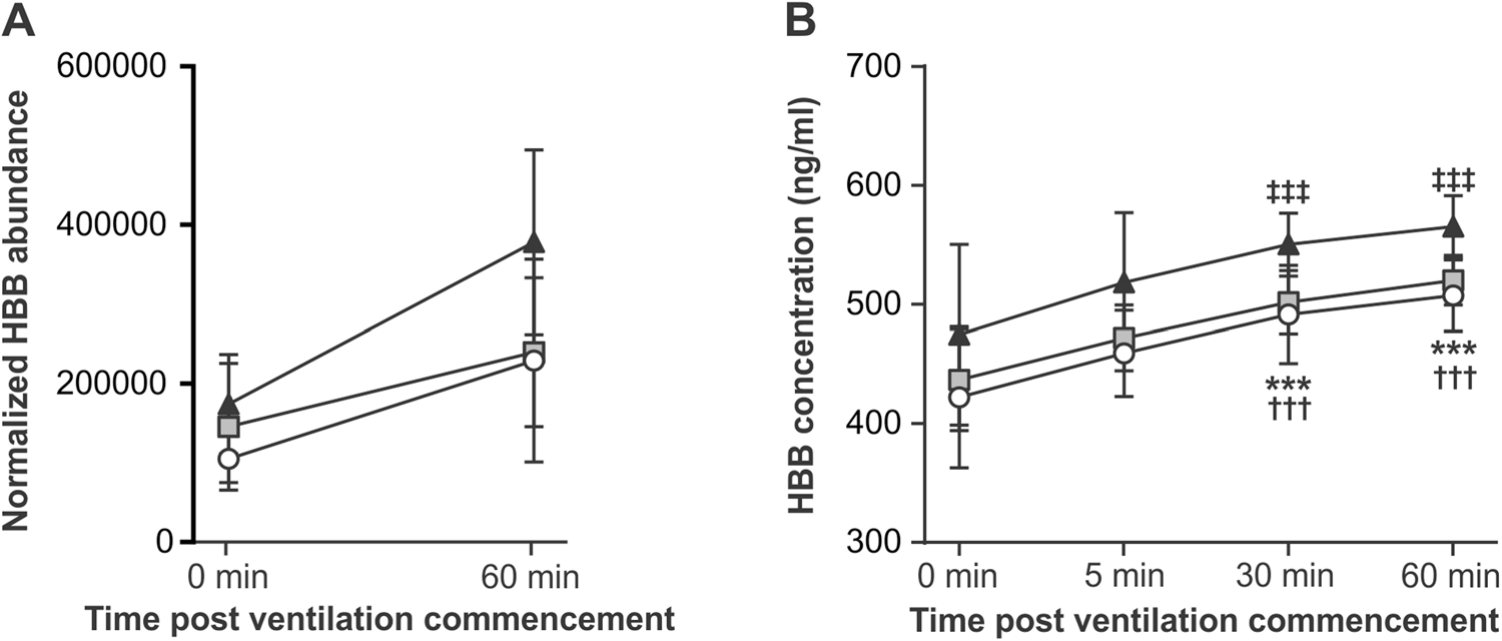
Normalized MS-SWATH HBB values (**A**) and ELISA-validation of plasma HBB concentration (**B**) in extremely preterm (open circle), preterm (grey square) and term (closed triangle) lambs during 60 minutes of ventilation. All data are expressed as mean ± SD. ^***^Differs from 0 minute concentration in extremely preterm group, *P* < 0.001 (one-way ANOVA with Tukey’s post tests), ^†††^Differs from 0 minute concentration in preterm group, *P* < 0.001 (one-way ANOVA with Tukey’s post tests), ^‡‡‡^Differs from 0 minute concentration in term group, *P* < 0.001 (one-way ANOVA with Tukey’s post tests). N = 5–7/group.

Mirroring the multi-variate analysis, 83% of differentially-expressed proteins were altered only in a single GA-group with the largest number of altered processes observed in the preterm group (20/40) when compared with the extremely preterm group (8/40) and term group (5/40), respectively. The hemoglobin proteins HBA2, HBB and HBBF were increased in all three GA-groups with increasing abundance of plasma HBB over-time in all GA-groups confirmed via ELISA (Fig. 2). COL1A2 protein abundance was increased in both the extremely preterm and term groups. FGB, HRG and RBP4 were decreased in both the extremely preterm group and preterm group, whilst TNC was decreased in the preterm and term groups.

### Ingenuity Pathway Analysis (IPA) identified GA-specific ventilation networks, enriched physiological functions and canonical pathways

**Figure 3:**
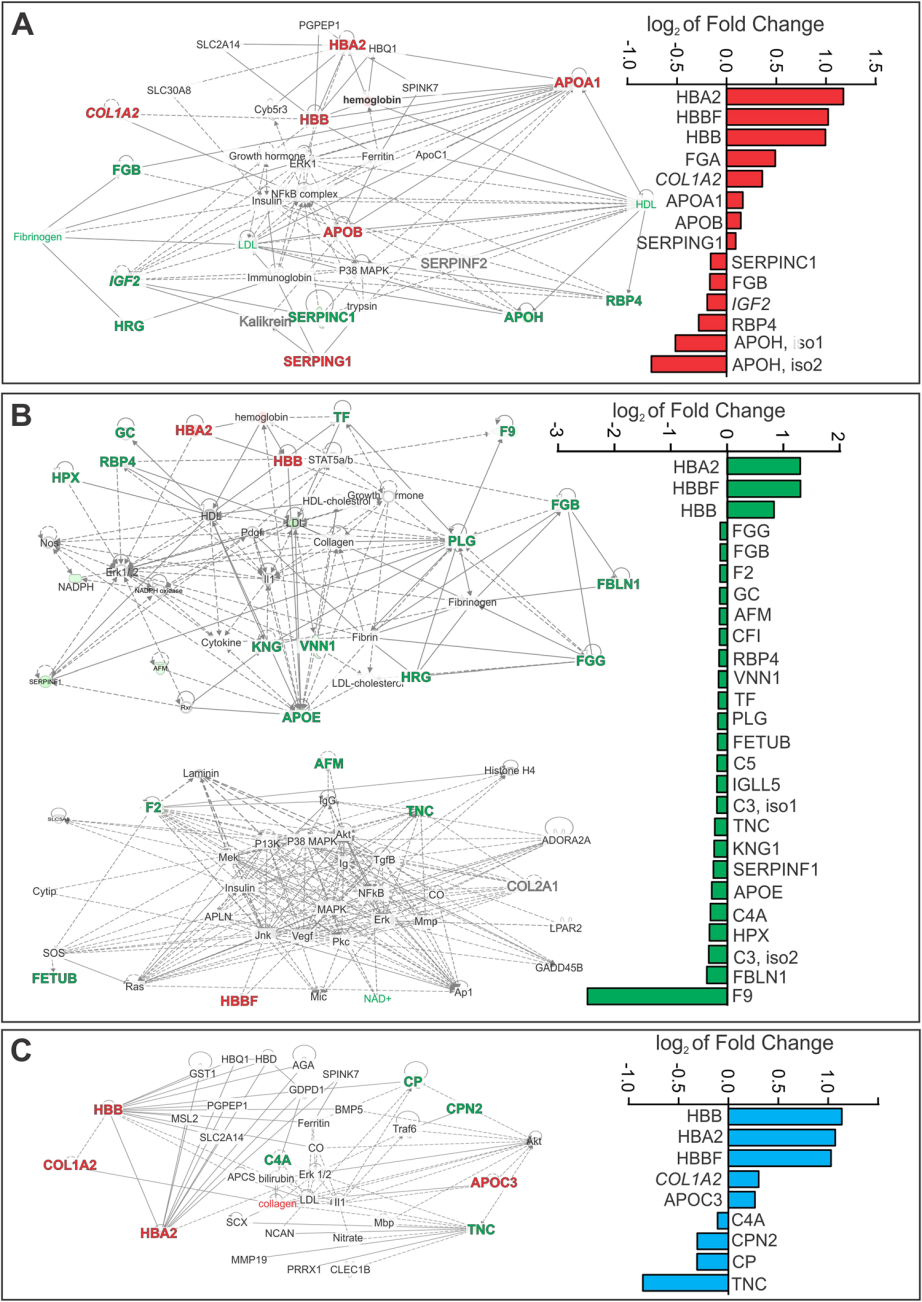
Network mapping of differential protein expression was GA-specific. Log_2_ fold changes and the GA-specific IPA-derived network pathways associated with differentially-expressed proteins in (**A**) extremely preterm, (**B**) preterm and (**C**) term ventilated lambs (P < 0.05, paired t-test pre-ventilatoin vs. 60 minute post-ventilation). Red and green nodes/text represent increased and decreased protein expressionat 60 minutes ventilation, relative to matched pre-ventilation samples. Proteins shown by white nodes were not identified in plasma samples. Solid lines indicate direct interactions or relationships, while dashed lines indicate indirect effects mediated by additional proteins. Proteins known to be influenced by antenatal betamethasone are shown in italics.

**Figure 4:**
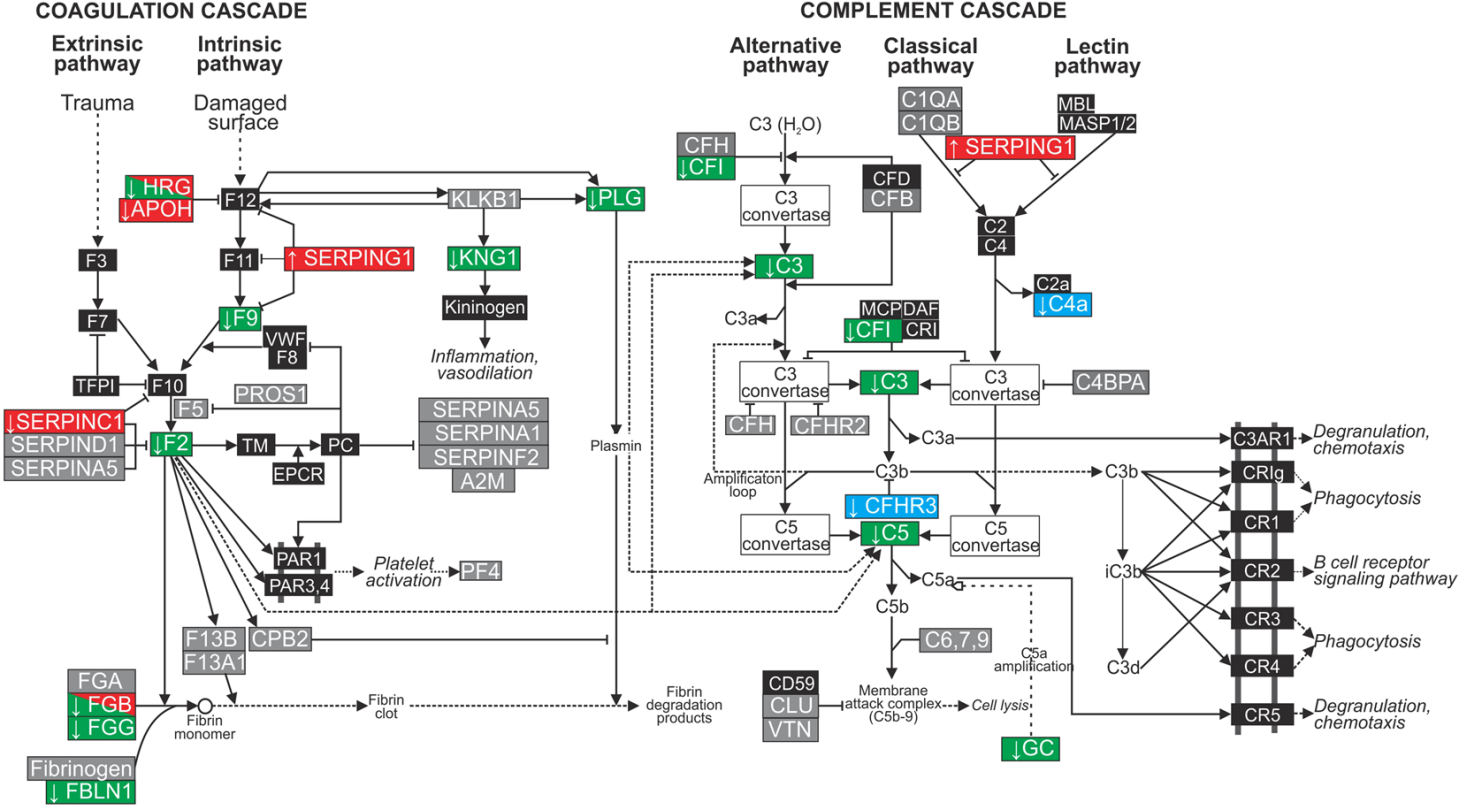
Alterations in protein expression predominantly mapped to the coagulation-complement cascade. Protein alterations observed in the extremely preterm (red), preterm (green) or term (blue) groups mapped to the coagulation-complement cascades. Grey boxes indicate identified proteins which were not altered by ventilation and black boxes represent proteins that were not identified in plasma samples. N = 7–8/group.

103/125 proteins (82.4%) qualified for inclusion in IPA-based analyses using a human homology threshold of ≥65% (Supplementary Table 2). 36/103 proteins (35.0%) exhibited differential expression in response to mechanical ventilation. GA-specific protein alterations and associated network pathway maps are detailed in Fig. 3. In the extremely preterm group, changes in protein expression were confined to a single network map and included increased expression of eight proteins and decreased expression of five. For six proteins the differential expression was specific to the extremely preterm group. The most prominent ventilation-induced alteration was observed in the preterm group, in which two network pathways were implicated covering differential expression of three increased proteins and 21 decreased proteins. Of note, 19 proteins were differentially expressed solely in the preterm group. Alterations in the term group were confined to a single network which included four increased proteins and four decreased proteins, with five proteins specifically altered in the term group. Considering all GA groups, 56% (20/36) of the differentially-expressed proteins belonged to the coordinated coagulation and complement cascades (Fig. 4).

IPA examination of significant canonical pathways associated with each GA-proteome revealed a unique association with the coagulation system canonical pathway in preterm lambs and a unique association with the complement system pathway in term lambs (Table 2). The canonical pathways for acute phase response signaling, LXR/RXR activation and FXR/RXR activation were identified as significant in all GA groups. The intrinsic prothrombin activation pathway was associated with proteome alterations detected in both extremely preterm and preterm lambs. In the molecular and cellular function analysis; small molecule biochemistry and cell-to-cell signaling and interaction functions were associated with all GA-groups. Lipid metabolism was significant to both preterm groups, and free radical scavenging functions and molecular transport function significant in the preterm and term groups (Table 2). Cellular movement were uniquely identified in the extremely preterm group alone. There were no shared physiological functions within the top 5 identifications (Table 2). Organismal development was significant in extremely preterm lambs alone, organismal function in preterm lambs and behavior and hematopoiesis functions identified in term lambs. Immune cell trafficking and cardiovascular system development and function and tissue morphology development and function were assigned to both the extremely preterm and preterm lamb groups.

**Table 2:** Identification of the top 5 enriched canonical pathways, molecular and cellular functions and physiological functions within the plasma proteome after 60 minutes of ventilation.

	Extremely preterm	Preterm	Term
Top canonical pathways	*Acute phase response signaling*	*Acute phase response signalling*	*LXR/RXR activation*
	*LXR/RXR activation*	*FXR/RXR activation*	Atherosclerosis signaling
	Atherosclerosis signaling	**Coagulation system**	*FXR/RXR activation*
	*FXR/RXR activation*	*LXR/RXR activation*	*Acute phase response signaling*
	Intrinsic prothrombin activation pathway	Intrinsic prothrombin activation pathway	**Complement system**
Molecular and cellular functions	Lipid metabolism	*Cell-to-cell signaling and interaction*	Cell death and survival
	*Small molecule biochemistry*	Free radical scavenging	Molecular transport
	Cell death and survival	*Small molecule biochemistry*	Free radical scavenging
	**Cellular movement**	Molecular transport	*Small molecule biochemistry*
	*Cell-to-cell signaling and interaction*	Lipid metabolism	*Cell-to-cell signaling and interaction*
Physiological system development and function	Organ morphology	Hematological system development and function	Organ morphology
	Immune cell trafficking	**Organismal functions**	**Behavior**
	Cardiovascular system development and function	Immune cell trafficking	**Connective tissue development and function**
	**Organismal development**	Cardiovascular system development and function	Hematological system development and function
	Tissue morphology	Tissue morphology	**Hematopoiesis**

IPA identified the top 5 enrichments based on their Fisher exact test p-value (P < 0.001 all analysis). Enrichments that were specific to a single GA-group are shown in bold, enrichments that are common to all GA-groups are italicized.

### GA-specific protein-function associations were identified within the plasma ventilation proteome

**Figure 5:**
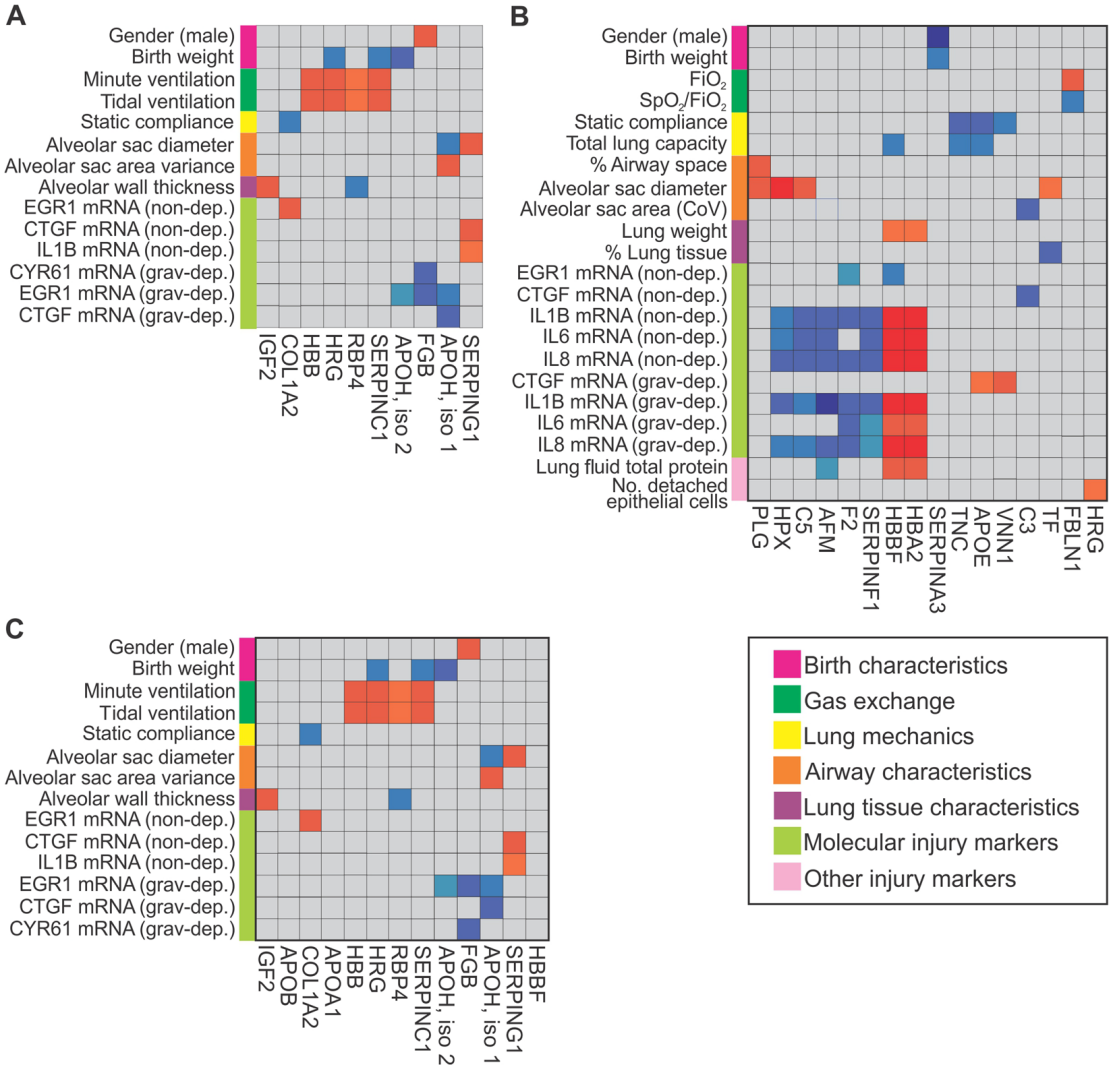
Unique protein-function associations were identified within each GA-group. CIM map representation of significant associations between changes in protein level measured prior to ventilation and at 60 minutes ventilation and functional outcomes in extremely preterm (**A**), preterm (**B**) or term (**C**) lambs. Red squares represent positive Pearson correlations (r ≥ 0.7) and blue squares represent negative Pearson correlations (r ≤ −0.7), with color intensity indicative of strength of correlation. Significance of correlations was calculated using a Mantel test with 1000 permutations. Non-significant results with r values of −0.69 to 0.69 are show in in grey. Functional outcomes have been grouped according to the colour key displayed. N = 7–8/group.

Correlation analysis identified protein-function associations specific to each gestation group (Fig. 5). In the extremely preterm group 4 differentially-expressed proteins (SERPINC1, RBP4, HRG and HBB) exhibited positive correlation against minute ventilation and tidal ventilation (Fig. 5A). Gene expression of injury markers EGR1/CTGF and EGR1/CYR61 in the gravity-dependent lung was negatively correlated against APOH and FGB respectively. Conversely gene expression of EGR1 and CTGF/IL1B in non-dependent lung was positively correlated against proteins COL1A2 and SERPING1. Proteins associated with birth characteristics (gender, birth weight) included FGB, APOH, SERPINC1 and HRG.

In the preterm group, the differential expression of 7 plasma proteins was either positively (HBA2, HBBF) or negatively (SERPINF1, F2, AFM, C5, HPX) associated with gene expression of inflammatory makrers in the non-dependent and gravity-dependent lung (Fig. 5B). Static compliance and total lung capacity were negatively associated with the proteins VNN1, FGG, TNC and SERPINA3, whilst FBLN1 was positively associated with FiO_2_ levels and negatively associated with the SpO_2_/FiO_2_ ratio.

The gas exchange parameters; SpO_2_/FiO_2,_ SpO_2_, OI, minute ventilation and tidal ventilation were positively associated with altered expression of proteins COL1A2 and CFHR3-like in term aniamls (Fig. 5C). CPN2, APOC3 and CP were negatively correlated with 6 gene markers of injury in the non-dependent lung and 2 gene markers in the gravity-dependent lung, however APOC3 also was positively associated with the birth characteristics ‘birth weight’.

## Discussion

Although essential for survival, mechanical ventilation is closely associated with a high risk of developing ventilation induced lung injury (VILI). Whilst the cause of VILI has been well studied and documented, the impact of gestational age (GA) at ventilation on VILI development has not been comprehensively studied. In the ventilated preterm lamb model 83% of differentially-expressed proteins identified by SWATH-MS were confined to a single GA-group, with the largest number of alterations observed within the preterm plasma proteome. Furthermore, correlation of differential protein expression against key measures of lung function and injury confirmed associstion between changes in protein expression and GA-specific biomarkers of the early lung injury response. Such tools may contribute to the development of individualized treatment strategies aimed at protecting the preterm lung and minimizing damage.

Consistent with clinical experience, increasing prematurity was associated with reduced oxygenation, impaired lung compliance and increasing evidence of lung injury. Paradoxically however, the greatest magnitude of differentially-expressed proteins were observed in the preterm group rather than the extremely preterm group. The answer to this paradox may lie in significantly different physiological responses to the same ventilation strategy. Amongst the functional parameters measured, only differences in gas exchange differentiated extremely preterm and preterm lambs. Lung injury is rarely consistent throughout the lung, exhibiting a regional gravitational behavior that is postulated to be initiated by ventilation and aeration homogeneity^24^. We have previously shown that the preterm group exhibits better dependent lung aeration in early life than the extremely preterm and term group^25^. The results of this study, in which the largest proteome alterations were observed in lambs in whom the dependent hemithorax was preferentially aerated suggest additional studies characterizing regional proteome alterations within lung tissue are warranted.

In pretern lambs, several lines of evidence confirmed the influence of ventilation on the inflammatory response including (i) increased abundance of the inflammatory-mediator apolipoprotein E (APOE), (ii) reduced abundance of key members of the complement cascade including complement components 3 (C3) and 5 (C5), complement factor I (CFI), and the C5 amplification mediator vitamin D binding protein (GC), (iii) results from the integrative analysis in which the ventilation-associated abundance of seven proteins (HBA2, HBBF, SERPINF1, F2, AFM, C5, HPX) was strongly correlated with the gene expression of inflammatory markers IL1B, IL6 and IL8 in both non-dependent and gravity-dependent lung tissue. APOE is expressed by alveolar epithelial cell type I cells and plays an important role in facilitating lipoprotein transport^26^ and regulating airway hyperactivity^27^. In the current study we observed a significant decrease in APOE protein expression following ventilation, concurrent with morphological evidence of lung injury and increased IL1B gene expression. Our results parallel those of the ApoE null mouse in which ApoE ablation is associated with hypersensitive pulmonary inflammatory responses^28^ and increased susceptibility to VILI development^29,30^. Similarly reduced levels of complement precursor proteins and functional activity have previously been observed in preterm infants with RDS^31^, however this is the first study to associate complement cascade disturbances with mechanical ventilation of the preterm lung.

The coagulation pathway was also highly perturbed in ventilated preterm lambs which is suggestive of a prothrombotic response to ventilation in this GA cohort. Our observation of decreased levels of coagulant Factor II (F2) and Factor IX (F9) together with decreased levels of fibrinolysis mediators FGB, FGG, FBLN1 and PLG concur with anticoagulant hypofunction previously described in infants with respiratory distress syndrome (RDS)^32,33^ where under pathologic conditions, such as following initiation of mechanical ventilation, the hemostatic balance favours thrombosis^34,35^ and fibrin deposition^36,37^. Fibrin deposition may then exacerbate lung injury via surfactant inactivation and reduced surfactant synthesis, ultimately disturbing gas exchange.

Similar to the preterm ventilated proteome, extremely preterm lambs exhibited evidence of hemostatic dysregulation with increased expression of plasma protease C1 inhibitor precursor (SERPINC1) and fibrinogen A (FGA) and decreased expression of antithrombin III and apolipoprotein H (APOH). In addition, our novel identification of increased APOA1 in ventilated extremely preterm lambs is also of interest. APOA1, a lipid transport protein^26,38,39^ has not previously been associated with either VILI or mechanical ventilation. We postulate that increased expression of APOA1 in the ventilated extremely preterm lamb model may represent a lung protective mechanism, as increased levels of APOA1 in mouse models of respiratory disease are associated with anti-inflammatory activity^40,41^, anti-fibrotic properties^42,43^ and the promotion of tight junction recovery^44^.

In addition to identifying proteins that uniquely defined the response to ventilation in each GA, we also identified within our study increases in the differential expression of fetal hemoglobin (HBBF), hemoglobin beta (HBB) and hemoglobin subunit alpha-1/2 (HBA2) across GA groups. HBB expression during ventilation was further validated via ELISA and shown to steadily increase with time on ventilation in all three GA-groups. The primary function of all three forms of hemoglobin is the transport of oxygen from the lung to various peripheral tissues, and therefore their comparable increase across all GA groups may predominantly reflect a response to oxygen delivery via mechanical ventilation, indicative of critical hemoglobin achievement within the ventilation parameters applied. Given recent reports by Ballard *et al*.^45^ of comparable fold changes in endotracheal aspirate expression of HBB, HBA2 and HBBF in a cohort of 37 infants with chronic lung disease, our evidence of plasma alteration of hemoglobin proteins warrants further investigation as a potential non-invasive monitoring tool of preterm lung injury.

Despite advances in neonatal care and continued research into therapeutic strategies aimed at prevention of BPD, the incidence of this condition has remained unchanged^46^. As such, a major finding with significant clinical implications is our observation of reduced protein abundance of retinol binding protein 4 (RBP4) in ventilated extremely preterm and preterm lambs. Vitamin A supplementation has been proposed as a lung protective therapy in preterm infants but results of clinical trials to date have been conflicting^47^. RBP4 transports retinol with a 1:1 ratio^48^, thus our finding of reduced RBP4 in ventilated preterm lambs may provide a biological rationale for the relatively modest improvement in respiratory outcomes observed following vitamin A delivery in very preterm infants.

Gaps in knowledge regarding the mechanisms underlying VILI are related, in part, to the challenges associated with studying an extremely preterm or preterm cohort within the confines of the Neonatal Intensive Care Unit. The preterm lamb model, with its ability to mirror the clinical setting of preterm birth^10,24,25,49–54^ and high sheep-human protein homology (82% of identified proteins exhibit high homology) is well suited to address this challenge, with some limitations. The ventilated preterm lamb model is labor-intensive and expensive, limiting the study to group sizes of 7–8 lambs/group and a single ventilation strategy. However, the techniques and workflow used in the current study provide the platform for larger studies focusing on longer periods of different mechanical ventilation strategies. Antenatal betamethasone exposure was limited to the two premature groups only, consistent with current clinical practice^16^ and the lack of steroid exposure in the term group may have influenced our results, specifically suppression of IGF2^55^ and COL1A2^56^ proteins. Whilst these studies suggest a limited influence of betamethasone on the observed proteome sets, confirmation via direct comparison of betamethasone treated and untreated samples may be warranted. Similarly the influence of anaesthesia has not been formally delineated, however up-stream analysis within IPA was unable to identify any association between the anaesthtic agents used (ketamine and midazalom) and the proteins identified. The inability to maintain survival in preterm lambs without any respiratory support limits the ability to determine whether the proteome differences were solely related to a ventilation response. Also, our investigation of the plasma proteome, whilst allowing for rapid clinical translation, is reflective of a whole body response and is therefore not confined to ventilation-associated alterations. To overcome this we excluded any proteins known to be related to the birth response or antenatal steroid exposure. Nevertheless, follow up studies in a sample type such as BALF, which directly reflects ventilation-induced damage within the lung, will be important.

Respiratory support is necessary to support preterm life, however, even brief periods of mechanical ventilation when applied to the immature lung can initiate lung injury. As a consequence, whilst advances in respiratory care have significantly altered survival, rates of chronic lung disease have not changed for 30 years^57^. If we are to improve the respiratory outcomes of those born prematurely, we must increase our understanding of the basic pathophysiology underlying VILI development. This is the first study to use proteomics and integrated bioinformatics to (i) identify the injury mechanisms and pathways which underlie VILI development and (ii) define the protein-function associations underlying VILI. By characterizing the extremely preterm and preterm VILI proteome, this study importantly broadens our understanding of how prematurity-specific factors, such as the developmental state of the lung, shape the cellular response to ventilation. Furthermore, our demonstration of clear differentiation between the ventilation-associated proteome of extremely preterm, preterm and term lambs in response to a uniform ventilation strategy supports a need for the development of individualized respiratory support approaches which are tailored to both the gestational age of the infant and their underlying pathology.

## Materials and Methods

### Antenatal Management

Date-mated Border-Leicester/Suffolk lambs, mated from the same flock over one winter, were delivered via caesarean section under general anesthesia at one of three gestational age (GA) cohorts: 119–120 d (extremely preterm), 127–128 d (preterm) or 139–140 d (term). These GAs are consistent with lung morphology in human infants at approximately 23 weeks, 27–28 weeks, and term gestation. Consistent with clinical practice^58^ the 119–120 d and 127–128 d cohorts were exposed to maternal 11.4 mg IM betamethasone 24 and 48 h before delivery. Whilst on placental support the carotid arterial and external jugular vein were cannulated, the trachea intubated with an appropriately sized cuffed endotracheal tube, and lung fluid passively drained^10,24^. After delivery, animals were weighed and placed supine, bedside monitoring commenced, intravenous fluids commenced (4% dextrose, 0.18% sodium chloride at 4–5 ml/hr) and mechanical respiratory support initiated. Continuous infusions of ketamine and midazolam were used to maintain anesthesia/analgesia and suppress spontaneous respiratory effort throughout the ventilatory period.

### Mechanical ventilation strategy and physiological monitoring

All lambs were ventilated for 60 min using the same strategy. Previous studies of this animal model have demonstrated alterations in gene expression and histological evidence of VILI after 15–30 min of ventilation^59–61^. Initially a sustained lung inflation was delivered at 40 cmH_2_O using a Neopuff Infant Resuscitator (Fisher Paykel Healthcare, Auckland, New Zealand) as per our previous reports^10,51,60^. Thereafter the lung was supported with positive pressure ventilation (SLE5000, SLE UK Ltd, South Croyden UK) in a volume-targeted ventilation mode (positive end-expiratory pressure 8 cmH_2_O, maximum positive inspiratory pressure 35 cmH_2_O, inspiratory time 0.4 s, initial rate 60 inflations per minute and initial tidal volume 7 ml/kg). To monitor physiological stability, preductal peripheral oxygen saturation (SpO_2_), heart rate, arterial blood pressure, and rectal temperature (HP48S monitor, Hewlett Packard) were recorded continuously from birth, and arterial blood gas analysis (pH, BE, PaO_2_, PaCO_2_) performed at 5 min of age and then 15 minutely from the first inflation. Parameters of oxygenation, including oxygenation index (OI) and alveolar-arterial oxygen difference (AaDO_2_), were calculated using standard formulae. FiO_2_, tidal volume (5.5 to 8 ml/kg) and ventilator rate (40 to 60) were adjusted as appropriate using a standardized protocol to maintain SpO_2_ between 88–95% and partial arterial pressure of carbon dioxide between 45–60 mmHg. At 60 minutes, all lambs were ventilated with an FiO_2_ of 1.0 for 3 min and disconnected to atmosphere for 2 min. An *in vivo* super syringe pressure–volume (PV) curve was obtained using a maximum pressure of 35 cmH_2_O to determine static respiratory system compliance (ml/kg/cmH_2_O). At the end of the study period all lambs received a lethal IV dose of pentobarbitone.

### Sample collection

Arterial blood samples for SWATH-MS proteomic analysis were collected at study commencement and following 60 minutes of ventilation in S-Monvette® tubes (Sarstedt), and were centrifuged at 3000 rpm, 10 minutes, 10 °C. Plasma was stored at −80 °C until required. Lambs were euthanized following mapping of the PV curve and the whole lung removed and weighed. Bronchoalveolar lavage fluid (BALF) was collected by repetitive saline lavage of the isolated left lung. Tissue samples from standardized non-dependent (ventral) regions and gravity-dependent (dorsal) regions of the right lower lung lobe were snap-frozen for RNA isolation. The right upper lobe was inflation fixed with 4% paraformaldehyde at 20 cmH_2_O and then paraffin embedded.

### Lung histology

Histological examination was performed on hematoxylin and eosin stained 4 μm formalin-fixed lung sections from three standardized lung regions. Post-staining, five fields of view (FOV)/region totalling 15 FOV/lamb were photographed (Leica Microsystems) and injury measurements performed using Image J software with % lung tissue, % airway space and amount of lung tissue (μm^2^) determined in 8-bit converted images using the threshold function to optically determine areas of tissue (grey) versus airway space (red). Measurements of alveolar morphology were measured in five alveolar spaces/micrograph (total 25 micrographs/lamb measured) as predetermined by a standardized grid overlay. The total number of detached epithelial cells/micrograph (total 25 micrographs/lamb measured) were counted using the ImageJ picker tool.

### RNA preparation and quantitative PCR

RNA was extracted from lung tissue using TRIzol, and 0.1 μg RNA was reverse-transcribed into complementary DNA (cDNA). Primers were designed using the Roche Universal ProbeLibrary Assay Design Center (Supplementary Table 2). qRT-PCR reactions (10 μl) contained 2.5 μl diluted cDNA, 5 μl FastStart TaqMan Probe Master (Roche), 900 nM of each primer and 250 nM of probe mix. All reactions were performed in triplicate on the Light-Cycler 480 System (Roche). The 2-ΔΔCt method^23^ was used to calculate relative changes in gene expression, determined from qRT-PCR experiments using GAPDH as a housekeeping gene and relative to the term group^62^.

### Protein detection in BALF samples

Lavage fluid was centrifuged at 300 g and 4 °C for 10 min, with determination of total protein concentration in the BALF supernatant using a commercially available modified Lowry assay kit (Thermo Fisher Scientific).

### Determination of plasma protein composition via SWATH-MS

#### Plasma sample preparation

Briefly, 25 μL of plasma sample was diluted in 475 μL of 50 mM ammonium bicarbonate solution before reducing with dithiothreitol (5 mM DTT) and alkylating with iodoacetamide (10 mM IAA). One fifth of the reduced and alkylated sample (121 μL) was digested with 10 μL trypsin (20 μg) for 16 hours at 37 °C. The digested sample was diluted (X 10.5) in 0.1% formic acid prior to all mass spectrometry analysis.

#### Information Dependent Acquisition Mass Spectrometry

A 5600 TripleTOF mass spectrometer (AB Sciex) coupled to an Eksigent Ultra nanoLC-1D system (Eksigent) was employed for both IDA and SWATH-MS analysis. For 1D IDA data, 10 μl of digested sample from each group was pooled, making three pooled groups (extremely preterm, preterm and term) and 5 μl duplicates of each pooled sample was injected. For 2D IDA 20 μl of each group pooled-sample was pooled (total 60 μl), then dried and fractionated in eight fractions using a High pH Reversed-Phase Peptide Fractionation kit (Pierce). 10 μl of each fraction was injected for 2D IDA.

#### SWATH Library Construction

Protein identification from IDA data was performed with ProteinPilot (v4.2; AB Sciex) based on a NCBI database, filtered for sheep and Ovis aries and containing 117933 protein entries. SWATH data were extracted using PeakView (v2.1) with peptides (max 100 peptides per protein) with confidence ≥99% and false discovery rate (FDR) ≤ 1% included in the quantitation and shared and modified peptides excluded. A local peptide assay library was derived from the 1D IDA and contained 163 identified proteins. An extended peptide assay library was constructed from the combination of the 1D and 2D IDA and contained 179 identified proteins.

#### Data independent acquisition (SWATH)

For SWATH-MS experiments 5 μl of each digested sample (total N = 22 samples) was injected in random order, with two technical replicates for each injection. SWATH data were acquired with one blank run between every sample.

#### SWATH Data Analysis

SWATH peaks were extracted using PeakView (AB Sciex; v2.1). Shared and modified peptides were excluded. Peak extraction parameters were set as the following: 100 peptides per protein, 6 transition ions per peptide, peptide confidence threshold 99%, FDR extraction threshold 1%, XIC (Extract Ion Chromatogram) retention time window 10 min and mass tolerance 75 ppm. The extracted transition ion peak areas, peptide peak areas and protein peak areas were exported in Excel for further statistical analysis.

### Validation of plasma hemoglobin beta (HBB) concentrations

HBB was detected in plasma samples obtained prior to ventilation and at 5, 30 and 60 minutes post ventilation via ELISA (Mybiosource Inc, San Diego, USA).

### Bioinformatics and Statistical Analysis

#### Physiological and functional parameters

Shapiro-Wilk normality testing confirmed the normality of data distribution, with results represented as either mean values with their standard deviation (SD) or median and interquartile range. Significant differences between gestation groups were sought using parametric and non-parametric ANOVA as appropriate (one-way ANOVA or Kruskall-Wallis ANOVA, respectively) with post hoc testing to identify intergroup differences as necessary (Tukey’s post hoc test or Dunn’s multiple comparison test, respectively). All statistical analysis was performed with GraphPad PRISM 6 (GraphPad Software, SanDiego, CA) and P < 0.05 considered significant. Unless otherwise stated P values refer to ANOVA.

#### Assessing proteomes that differed between the GA groups

To assess whether extremely preterm, preterm and term groups exhibited compositional proteome differences following 60 minutes of ventilation, we firstly used an unsupervised clustering approach (principal component analysis; PCA) based on scaled protein abundances (ie. difference between post- and pre-ventilation protein abundance). To further investigate which proteins potentially contribute most to differences between GA-groups, we used partial least squares discriminant analysis (PLS-DA) in the R package MixOmics (version 3.1.1).

#### Determination of differential protein expression

To identify significant alterations in protein abundance paired 0 and 60 minute plasma samples were compared first by a paired t-test and represented within the results as log_2-_transformed expression. P values of <0.05 were considered significant.

#### Ingenuity Pathway Analysis (IPA)

To address the requirement for human, mouse or rat data in IPA, we first used the NCBI Basic Local Alignment Search Tool (BLAST) to identify those sheep proteins with ≥65% homology to human protein sequences. These highly homologous protein identities were then uploaded into Qiagen’s IPA system for core analysis and down-stream processing to identify canonical pathways, disease and functions and protein networks that were most significant within each GA group.

Correlation between differential protein abundance and functional parameters: To test how differential protein abundance and clinical and functional parameters co-vary within the gestation groups, distance matrices using Manhattan distances were calculated for proteins (60 minute abundance − 0 minute abundance) and functional parameters separately, and then Pearson correlation coefficients between both distance matrices were calculated. Significance of correlations was calculated using a Mantel test with 1000 permutations.

### Study approval

All experiments were conducted according to the guidelines of the National Health and Medical Research Council (Australia), with prior approval from the Animal Ethics Committee of the Murdoch Children’s Research Institute (Melbourne, Australia).

## Electronic supplementary material

Table 1 and 2

## Data Availability

The datasets generated during and/or analysed during the current study are available from the corresponding author on reasonable request.
