# Supplementary material for: Plasma proteomics reveals gestational age-specific responses to mechanical ventilation and identifies the mechanistic pathways that initiate preterm lung injury
Source: Sci Rep. 2018 Aug 22;8:12616. doi: 10.1038/s41598-018-30868-x (PMC6105628; doi:10.1038/s41598-018-30868-x)
Supplement: Supplementary file 1 — Table 1 and 2 [file 41598_2018_30868_MOESM1_ESM.docx]

**Plasma proteomics reveals gestational age-specific responses to mechanical ventilation and identifies the mechanistic pathways that initiate preterm lung injury**

Prue. M. Pereira-Fantini^1,2^*, Sean G. Byars^3,4^, Karen. E. McCall^1,5^, , Elizabeth. J. Perkins^1^, Regina. B. Oakley^1^, R. L. Dellacà^6^, Peter. A. Dargaville^7^, Peter. G. Davis^1,8,9^, Vera Ignjatovic^2,10^, David. G. Tingay^1,2,11^

^1^Neonatal Research, Murdoch Childrens Research Institute, Parkville, Australia.

^2^Department of Paediatrics, University of Melbourne, Parkville, Australia.

^3^Department of Pathology, University of Melbourne, Parkville, Australia.

^4^Centre for Systems Genomics, University of Melbourne, Parkville, Australia.

^5^University College Dublin, Dublin, Ireland.

^6^Laboratorio di Tecnologie Biomediche, Dipartimento di Elettronica, Informazione e Ingegneria Biomedica-DEIB, Politecnico di Milano University, Milano, Italy.

^7^Menzies Institute for Medical Research, University of Tasmania, Hobart, Australia.

^8^The Royal Women's Hospital, Parkville, Australia.

^9^Department of Obstetrics and Gynaecology, University of Melbourne, Parkville, Australia.

^10^Haematology Research, Murdoch Childrens Research Institute, Parkville, Australia.

^11^Department of Neonatology, Royal Children’s Hospital, Parkville, Australia.

*Corresponding author: Dr Prue M. Pereira-Fantini

Neonatal Research,

Murdoch Childrens Research Institute,

Royal Children’s Hospital,

Flemington Road,

Parkville, VIC 3052, Australia

Email: prue.pereira@mcri.edu.au

Phone: +613 8341 6452

**Supplementary Table 1: Clinical characteristics of study population at birth.** Parametric data are represented as means ± SD (parametric) and non-parametric data as median (interquartile range). P < 0.05 for comparison of: *extremely preterm vs preterm; †extremely preterm vs term; and ‡preterm vs term groups as determined by parametric or non-parametric ANOVA as appropriate (one-way ANOVA or Kruskall-Wallis ANOVA, respectively) with post hoc testing to identify intergroup differences (Tukey’s post hoc test or Dunn’s multiple comparison test, respectively).

|  | **Extremely preterm** | **Preterm** | **Term** |
| --- | --- | --- | --- |
| Number | 7 | 8 | 7 |
| Fetal lung fluid (ml/kg) | 18.5 ± 4.8 | 19.6 ± 5.1 | 18.6 ± 7.1 |
| Birth weight (kg) | 2.43 ± 0.25^*†^ | 3.26 ± 0.22^‡^ | 4.85 ± 0.33 |
| Sex ratio (M:F) | 4:3 | 5:3 | 5:2 |
| Fetal arterial pH | 7.34 (7.31, 7.36) | 7.36 (7.36, 7.38) | 7.31 (7.29, 7.35) |
| Fetal arterial base excess | 3.9 ± 2.1 | 2.2 ± 2.6 | 0.5 ± 3.7 |
| Fetal partial pressure of O_2_ (mmHg) | 24.9 ± 3.9 | 26.0 ± 2.0 | 24.4 ± 2.0 |
| Fetal partial pressure of CO_2_ (mmHg) | 56.8 ± 5.1 | 48.9 ± 3.3 | 54.7 ± 6.7 |

**Supplementary Table 2: Plasma protein composition via SWATH-MS detection**. Mean log_2_ fold change (log_2_ FC) in protein expression and P value (paired t-test) between matched plasma samples obtained prior to ventilation and following 60 minutes of ventilation from lambs delievered extremely preterm (119-120 days; n=7), preterm (127-128 days; n=8) or term (139-140 days; n=7). Unchanged proteins are denoted in grey boxes and differentially-expressed proteins in white. ^†^Identification is predicted by automated computational analysis derived from a genomic sequence using the Gnomon gene prediction method, supported by expressed sequence tag (EST) evidence. ^*^Differentially-expressed proteins with previous demonstrated asociation in studies of acute lung injury in prematurity (bronchopulmonary dysplasia; BPD or respiratory distress syndrome; RDS) or adult disease (acute lung injury; ALI or acute respiratory distress syndrome;ARDS). Proteins for which expression is known to be influenced by antenatal betamethasone administration are shown in italics.

|  |  |  |  | **Extremely preterm** | | **Preterm** | | **Term** | |
| --- | --- | --- | --- | --- | --- | --- | --- | --- | --- |
| **Accession Number** | **Protein Identification** | **Gene name** | **Human homology (%)** | **log_2_ FC (mean)** | **P value** | **log_2_ FC (mean)** | **P value** | **log_2_ FC (mean)** | **P value** |
| XP_012005880 | Alpha-1B-glycoprotein isoform X2^†^ | A1BG | 60 | 0.0182 | 0.6473 | -0.0609 | 0.4620 | 0.0678 | 0.8010 |
| XP_012026960 | Alpha-2-macroglobulin isoform X2^†^ | A2M, iso 2 | 78 | -0.0863 | 0.5877 | -0.2123 | 0.0550 | 0.0061 | 0.9389 |
| XP_012026962 | Alpha-2-macroglobulin isoform X4^†^ | A2M, iso4 | 78 | -0.2262 | 0.1822 | -0.0630 | 0.8255 | -0.0119 | 0.9877 |
| NP_001009784 | Actin, cytoplasmic 1 | ACTG1 | 99 | -0.1606 | 0.8517 | -0.1647 | 0.9555 | -0.0537 | 0.9046 |
| XP_004009953 | Afamin isoform X1^†^ | AFM | 75 | -0.0802 | 0.3507 | -0.1307 | 0.0210 | -0.0206 | 0.9845 |
| XP_011990894 | Alpha-fetoprotein^†^ | AFP | 78 | -0.1298 | 0.2631 | -0.0648 | 0.2281 | -0.0400 | 0.6713 |
| XP_011989285 | Angiotensinogen^†^ | AGT | 64 | -0.0042 | 0.9336 | -0.1374 | 0.0955 | 0.0123 | 0.8699 |
| NP_001009802 | Alpha-2-HS-glycoprotein precursor | AHSG | 64 | 0.0496 | 0.2315 | -0.0364 | 0.1691 | 0.0166 | 0.5843 |
| 4LUH_A | Chain A, Complex Of Ovine Serum Albumin With 3,5-diiodosalicylic Acid | ALB | 75 | -0.0107 | 0.7178 | 0.0247 | 0.0817 | -0.0083 | 0.6141 |
| XP_012007629 | Protein AMBP^†^ | AMBP | 78 | -0.0747 | 0.8992 | -0.0037 | 0.8847 | -0.0255 | 0.7822 |
| XP_011973043 | Apolipoprotein A-I^* †^ | APOA1 | 79 | 0.1632 | 0.0297 | 0.0054 | 0.8207 | 0.0131 | 0.8952 |
| XP_012023502 | Apolipoprotein A-II^†^ | APOA2 | 72 | -0.0497 | 0.8288 | 0.1328 | 0.1073 | -0.0367 | 0.9793 |
| XP_011973037 | Apolipoprotein A-IV^†^ | APOA4 | 79 | -0.1302 | 0.2728 | -0.1645 | 0.0831 | 0.0046 | 0.9745 |
| XP_012031328 | Apolipoprotein B-100 isoform X1^* †^ | APOB | 74 | 0.1424 | 0.0019 | 0.0164 | 0.8475 | 0.0873 | 0.1977 |
| XP_011983584 | Apolipoprotein C-II isoform X2 ^†^ | APOC2 | 64 | 0.3461 | 0.2029 | 0.0963 | 0.3224 | 0.1861 | 0.4617 |
| XP_004016097 | Apolipoprotein C-III isoform X1^†^ | APOC3 | 70 | 0.1597 | 0.2850 | -0.0135 | 0.8753 | 0.2648 | 0.0340 |
| XP_011995868 | Apolipoprotein D^†^ | APOD | 83 | 0.0249 | 0.7699 | -0.1820 | 0.1158 | -0.1150 | 0.3494 |
| XP_011950465 | Aapolipoprotein E isoform X1^* †^ | APOE | 69 | -0.2584 | 0.1626 | -0.2697 | 0.0142 | -0.1350 | 0.2246 |
| XP_012016040 | Apolipoprotein F-like^†^ | APOF-like | 42 | -0.0009 | 0.9023 | -0.0924 | 0.2183 | -0.0003 | 0.6878 |
| XP_004013204 | Beta-2-glycoprotein 1^*^ ^†^ | APOH | 83 | -0.5173 | 0.0066 | 0.0513 | 0.5394 | 0.0358 | 0.8474 |
| XP_012000740 | eta-2-glycoprotein 1^* †^ | APOH | 83 | -0.7586 | 0.0318 | -0.7054 | 0.3109 | -0.2520 | 0.6074 |
| XP_012018769 | Apolipoprotein M isoform X2^†^ | APOM | 89 | 0.0572 | 0.5740 | -0.1054 | 0.6735 | -0.1648 | 0.1045 |
| XP_011996145 | ADP-ribosyl cyclase/cyclic ADP-ribose hydrolase 2 isoform X2^†^ | BST1 | 80 | 0.1263 | 0.7763 | 0.1533 | 0.9406 | -0.0187 | 0.7053 |
| XP_011970833 | Complement C1q subcomponent subunit A^†^ | C1QA | 73 | 0.0179 | 0.9352 | 0.1006 | 0.4728 | -0.0447 | 0.6681 |
| XP_011970830 | Complement C1q subcomponent subunit B^†^ | C1QB | 73 | 0.5411 | 0.1106 | 0.2300 | 0.3208 | 0.0667 | 0.9364 |
| AAB92374 | Complement component C3, partial | C3 | 81 | 0.0199 | 0.9196 | -0.1410 | 0.3062 | 0.0653 | 0.1925 |
| XP_004022870 | Complement C3 isoform X1, partial^*, †^ | C3, iso 1 | 79 | 0.0073 | 0.9724 | -0.1762 | 0.0137 | -0.0246 | 0.6596 |
| XP_004022911 | Complement C3-like ^†^ | C3-like | 73 | 0.0096 | 0.8766 | 0.0473 | 0.7247 | 0.0120 | 0.8120 |
| XP_004022959 | Complement C3-like, partial^†^ | C3-like | 70 | -0.0241 | 0.8248 | -0.0782 | 0.0789 | -0.0260 | 0.4302 |
| XP_011963503 | Complement C3-like^*, †^ | C3-like | 84 | -0.7257 | 0.4163 | -0.3246 | 0.0358 | -0.1430 | 0.4399 |
| XP_011956845 | Complement C4-A-like^*, †^ | C4A-like | 81 | -0.0823 | 0.3525 | -0.1697 | 0.1515 | -0.1098 | 0.0379 |
| XP_004013628 | C4b-binding protein alpha chain isoform X3^†^ | C4BPA, iso 3 | 60 | -0.5408 | 0.3710 | -0.0397 | 0.3725 | 1.1670 | 0.1612 |
| XP_004013629 | Apolipoprotein R-like isoform X1^†^ | C4BPA | 54 | -0.0403 | 0.6719 | -0.1361 | 0.1436 | -0.0398 | 0.7007 |
| XP_004004015 | Complement C5^†^ | C5 | 81 | -0.0167 | 0.9429 | -0.1876 | 0.1851 | 0.0212 | 0.9242 |
| XP_011981396 | Complement C5^*, †^ | C5 | 81 | 0.1622 | 0.1615 | -0.1722 | 0.0468 | 0.0043 | 0.9436 |
| XP_012012132 | Complement component C6^†^ | C6 | 80 | -0.4448 | 0.1380 | -0.2983 | 0.3300 | 0.0196 | 0.7461 |
| XP_012012133 | Complement component C7 isoform X1^†^ | C7 | 81 | -0.2764 | 0.2793 | 0.0852 | 0.8931 | -0.1852 | 0.3585 |
| XP_011985154 | Complement component C9^†^ | C9 | 69 | -0.0016 | 0.9574 | -0.0807 | 0.3148 | 0.0114 | 0.8209 |
| NP_001155360 | Complement component C1q receptor precursor | CD93 | 67 | -0.0256 | 0.9261 | -0.2979 | 0.5037 | -0.0050 | 0.9754 |
| XP_011965793 | Cadherin-5 isoform X2^†^ | CDH5 | 80 | 0.0409 | 0.7304 | -0.0646 | 0.9391 | -0.3970 | 0.1522 |
| XP_012018834 | Complement factor B^†^ | CFB | 81 | -0.0783 | 0.2081 | -0.0987 | 0.0960 | -0.0189 | 0.7311 |
| ADF57191 | Factor H, partial | CFH | 63 | -0.0097 | 0.9661 | -0.0463 | 0.4621 | 0.2455 | 0.4515 |
| XP_012042386 | Complement factor H isoform X1^†^ | CFH, iso 1 | 47 | 0.1211 | 0.3595 | 0.2053 | 0.6225 | -0.0763 | 0.6669 |
| XP_012028511 | Complement factor H isoform X4^†^ | CFH, iso 4 | 55 | 0.6819 | 0.0928 | 0.1049 | 0.6563 | -0.4110 | 0.7103 |
| XP_004013976 | Complement factor H-related protein 2^†^ | CFHR2 | 58 | -0.0198 | 0.7161 | -0.1063 | 0.1420 | -0.0099 | 0.9356 |
| XP_012028601 | Complement factor H-like^†^ | CFH-like | 58 | -0.1027 | 0.1621 | -0.0048 | 0.9433 | -0.1181 | 0.2071 |
| XP_012028255 | Complement factor H-related protein 3-like isoform X2^†^ | CFHR3-like | 57 | -0.1841 | 0.4366 | -0.4516 | 0.1386 | -0.3016 | 0.0466 |
| XP_011994196 | Complement factor I^*, †^ | CFI | 65 | -0.0957 | 0.3373 | -0.1321 | 0.0331 | -0.0350 | 0.7286 |
| XP_011969773 | Tetranectin^†^ | CLEC3B | 84 | -0.0823 | 0.5913 | -0.2617 | 0.0826 | -0.1374 | 0.5003 |
| XP_012005113 | Clusterin^†^ | CLU | 71 | 0.0265 | 0.7005 | -0.0375 | 0.7785 | 0.4245 | 0.2814 |
| XP_011983016 | Collagen alpha-1(I) chain isoform X4^†^ | COL1A1 | 97 | -0.5844 | 0.0673 | -0.0514 | 0.7727 | -0.1020 | 0.5775 |
| *XP_012001957* | *Collagen alpha-2(I) chain isoform X2*^*,^ *^†^* | *COL1A2* | *93* | *0.3587* | *0.0119* | *-0.1395* | *0.5392* | *0.3025* | *0.0149* |
| XP_011984071 | Cartilage oligomeric matrix protein isoform X2^†^ | COMP | 92 | 0.0920 | 0.5126 | -0.3695 | 0.0887 | -0.0574 | 0.5705 |
| XP_011999073 | Ceruloplasmin isoform X2^*, †^ | CP | 86 | -0.0348 | 0.4125 | -0.1732 | 0.0765 | -0.3154 | 0.0269 |
| XP_012039800 | Carboxypeptidase B2 isoform X2^†^ | CPB2 | 73 | -0.1416 | 0.5283 | -0.0133 | 0.9543 | 0.1819 | 0.4087 |
| XP_011975129 | Carboxypeptidase N catalytic chain^†^ | CPN1 | 86 | -0.0028 | 0.8813 | -0.0844 | 0.6643 | 0.0257 | 0.7982 |
| XP_011995841 | Carboxypeptidase N subunit 2^†^ | CPN2 | 75 | -0.5029 | 0.1172 | -0.1245 | 0.7055 | -0.3124 | 0.0290 |
| XP_012013901 | C-reactive protein^†^ | CRP | 69 | -0.0687 | 0.3577 | -0.2771 | 0.0662 | -0.0587 | 0.7324 |
| XP_011972688 | Cystatin-M^†^ | CST6 | 75 | -0.0517 | 0.5184 | 0.5205 | 0.5203 | -0.1706 | 0.3559 |
| XP_011983922 | Coagulation factor XIII A chain^†^ | F13A1 | 87 | 0.2502 | 0.2681 | -0.1661 | 0.6435 | -0.2044 | 0.4553 |
| XP_012007295 | Coagulation factor XIII B chain isoform X2^†^ | F13B | 76 | 0.0884 | 0.7600 | -0.5692 | 0.0559 | -0.0589 | 0.3912 |
| NP_001159667 | Prothrombin precursor^*^ | F2 | 81 | -0.0591 | 0.5624 | -0.1246 | 0.0177 | 0.0074 | 0.9899 |
| XP_011966845 | Coagulation factor V isoform X2^†^ | F5 | 70 | 0.0303 | 0.9017 | -0.2596 | 0.1661 | 0.1444 | 0.8658 |
| XP_012024806 | Coagulation factor IX^†^ | F9 | 85 | -0.6799 | 0.6478 | -2.4780 | 0.0431 | 0.5798 | 0.7006 |
| XP_012001366 | Fibulin-1 isoform X2^*, †^ | FBLN1 | 90 | 0.0117 | 0.9494 | -0.3566 | 0.0165 | -0.0922 | 0.6036 |
| XP_011997866 | Fetuin-B^†^ | FETUB | 66 | -0.1469 | 0.1607 | -0.1636 | 0.0116 | -0.0616 | 0.3429 |
| XP_004017232 | Fibrinogen alpha chain isoform X1^†^ | FGA |  | -0.0885 | 0.7647 | 0.2261 | 0.7620 | 0.1432 | 0.6170 |
| XP_012006312 | Fibrinogen beta chain^*, †^ | FGB | 82 | -0.1686 | 0.0463 | -0.1194 | 0.0246 | -0.0694 | 0.3195 |
| XP_012006314 | Fibrinogen gamma chain isoform X1 ^†^ | FGG | 82 | -0.1266 | 0.1442 | -0.1192 | 0.0042 | -0.0412 | 0.4275 |
| XP_004004957 | Fibronectin isoform X8^†^ | FN1 | 93 | -0.0111 | 0.8329 | -0.0847 | 0.1317 | -0.0375 | 0.4550 |
| XP_011990876 | Vitamin D-binding protein^*, †^ | GC | 82 | -0.0614 | 0.4076 | -0.1269 | 0.0441 | -0.0011 | 0.9615 |
| XP_011968829 | Glutathione peroxidase 3 isoform X2^†^ | GPX3 | 88 | 0.3052 | 0.3290 | -0.1602 | 0.2950 | 0.0312 | 0.8791 |
| NP_001232935 | Gelsolin | GSN | 100 | -0.1007 | 0.2816 | -0.0432 | 0.2825 | 0.0360 | 0.1018 |
| XP_011983870 | Hyaluronan-binding protein 2 isoform X2^†^ | HABP2 | 82 | -0.7840 | 0.1369 | -1.0040 | 0.1768 | -0.1944 | 0.7757 |
| XP_011999574 | Hemoglobin subunit alpha-1/2 isoform X2^†^ | HBA2 | 87 | 1.1760 | 0.0011 | 1.3010 | 0.0158 | 1.0710 | 0.0097 |
| ABC86527 | Hemoglobin subunit beta^*^ | HBB | 99 | 0.9954 | 0.0302 | 0.8352 | 0.0070 | 1.1380 | 0.0065 |
| XP_012019955 | Hemoglobin fetal subunit beta^*, †^ | HBBF | 78 | 1.0220 | 0.0016 | 1.3050 | 0.0132 | 1.0290 | 0.0127 |
| XP_011982217 | Hepatocyte growth factor activator isoform X6^†^ | HGFAC | 81 | -0.1268 | 0.4740 | 0.0040 | 0.7789 | 0.0404 | 0.7473 |
| XP_004016259 | Hemopexin isoform X1^*, †^ | HPX | 73 | -0.0903 | 0.2145 | -0.3095 | 0.0057 | -0.0823 | 0.3688 |
| XP_004003109 | Histidine-rich glycoprotein^†^ | HRG | 62 | -0.1918 | 0.0112 | -0.1608 | 0.0343 | -0.0244 | 0.7868 |
| *XP_011956934* | *Insulin-like growth factor II isoform X1*^*,^ *^†^* | *IGF2* | *83* | *-0.1967* | *0.0279* | *-0.2028* | *0.1808* | *-0.2607* | *0.0995* |
| XP_011982590 | Cation-independent mannose-6-phosphate receptor isoform X2^†^ | IGF2R | 80 | -0.4698 | 0.8900 | -0.1594 | 0.9395 | -0.6119 | 0.4580 |
| NP_001009436 | Insulin-like growth factor-binding protein 2 precursor | IGFBP2 | 88 | -0.2438 | 0.2183 | -0.2131 | 0.1901 | 0.0275 | 0.8064 |
| AAB33382 | Insulin-like growth factor-binding protein-4, partial | IGFBP4 | 96 | -0.4551 | 0.1168 | 0.2737 | 0.0795 | -0.0573 | 0.5384 |
| B30554 | Ig lambda chain C region - sheep (fragment) | IGLL5 | 71 | -0.1902 | 0.3928 | 0.0781 | 0.9471 | -0.7433 | 0.1590 |
| XP_011977256 | Inter-alpha-trypsin inhibitor heavy chain H1^†^ | ITIH1 | 82 | -0.0099 | 0.9763 | -0.0482 | 0.3457 | 0.0059 | 0.8118 |
| XP_012002739 | Inter-alpha-trypsin inhibitor heavy chain H2^†^ | ITIH2 | 86 | -0.0902 | 0.1330 | -0.0549 | 0.2162 | -0.0174 | 0.8515 |
| XP_011977257 | Inter-alpha-trypsin inhibitor heavy chain H3^†^ | ITIH3 | 87 | -0.0428 | 0.3026 | 0.0101 | 0.9507 | -0.0504 | 0.5242 |
| XP_011977244 | Inter-alpha-trypsin inhibitor heavy chain H4 isoform X3^†^ | ITIH4 | 74 | -0.0714 | 0.1409 | 0.0079 | 0.6323 | -0.0316 | 0.4354 |
| XP_011961174 | Plasma kallikrein isoform X2^†^ | KLKB1 | 78 | 0.0957 | 0.4659 | -0.2836 | 0.3658 | -0.0855 | 0.7618 |
| XP_011997864 | Kininogen-1 isoform X2^*, †^ | KNG1 | 76 | -0.0223 | 0.7680 | -0.2267 | 0.0377 | -0.0938 | 0.5660 |
| XP_011999269 | L-lactate dehydrogenase C chain isoform X1^†^ | LDHC | 86 | -0.1691 | 0.5534 | -0.1931 | 0.5820 | 0.2941 | 0.6604 |
| XP_011994950 | Leucine-rich alpha-2-glycoprotein^†^ | LRG1 | 69 | 0.0328 | 0.9043 | -0.1797 | 0.0737 | 0.0006 | 0.6060 |
| XP_011979193 | Lumican^†^ | LUM | 89 | 0.0973 | 0.6159 | -0.0160 | 0.6887 | 0.0428 | 0.7051 |
| XP_012007641 | Alpha-1-acid glycoprotein 1 isoform X1^†^ | ORM1 | 57 | -0.0209 | 0.5017 | 0.1181 | 0.2375 | -0.1128 | 0.6733 |
| XP_011990905 | Platelet factor 4 isoform X2^†^ | PF4 | 70 | 0.0590 | 0.9050 | -0.5945 | 0.4100 | -0.1204 | 0.6891 |
| XP_012022145 | N-acetylmuramoyl-L-alanine amidase^†^ | PGLYRP2 | 72 | -0.0401 | 0.9497 | -0.1844 | 0.5472 | -0.1587 | 0.2396 |
| XP_012038601 | Plasminogen isoform X1^*, †^ | PLG | 79 | -0.1119 | 0.2927 | -0.1578 | 0.0383 | -0.0184 | 0.7308 |
| XP_011998676 | Vitamin K-dependent protein S isoform X2^†^ | PROS1 | 82 | 0.0709 | 0.8136 | -0.2039 | 0.1815 | -0.1024 | 0.2841 |
| XP_011984967 | Protein HP-20 homolog isoform X2^†^ | HP-20 | 0 | -0.1021 | 0.5027 | -0.2958 | 0.1307 | -0.0675 | 0.4053 |
| XP_011984966 | Protein HP-25 homolog 2 isoform X2^†^ | HP-25 | 0 | -0.0563 | 0.8011 | -0.2607 | 0.0618 | -0.0313 | 0.3323 |
| XP_011992224 | ,Retinol-binding protein 4 isoform X2^*, †^ | RBP4 | 92 | -0.2804 | 0.0041 | -0.1404 | 0.0192 | -0.0563 | 0.9457 |
| XP_011953679 | Alpha-1-antiproteinase isoform X1^†^ | SERPINA1 | 70 | -0.0500 | 0.6503 | -0.1441 | 0.0535 | -0.0976 | 0.3215 |
| XP_004022991 | Serpin A3-5-like isoform X1, partial^†^ | SERPINA3 | 62 | -0.3585 | 0.5265 | -0.0271 | 0.5653 | 0.0421 | 0.6339 |
| XP_011963751 | Serpin A3-1-like^†^ | SERPINA3 | 61 | 0.0817 | 0.2691 | -0.3271 | 0.1915 | 0.0042 | 0.4106 |
| XP_012024052 | Serpin A3-5-like isoform X3^†^ | SERPINA3 | 64 | 0.0802 | 0.5693 | 0.4516 | 0.8825 | 0.3350 | 0.2813 |
| XP_012027973 | Serpin A3-7-like isoform X3^*, †^ | SERPINA3 | 63 | -0.0559 | 0.5623 | -0.1330 | 0.0038 | -0.0368 | 0.8626 |
| XP_004018025 | Plasma serine protease inhibitor^†^ | SERPINA5 | 87 | 0.0585 | 0.6975 | -0.0644 | 0.6146 | -0.0889 | 0.5941 |
| XP_012024060 | Corticosteroid-binding globulin^†^ | SERPINA6 | 69 | -0.5546 | 0.1246 | -0.4237 | 0.0759 | -0.0864 | 0.5919 |
| XP_011961522 | Thyroxine-binding globulin isoform X1^†^ | SERPINA7 | 83 | -0.0245 | 0.6279 | 0.0038 | 0.6717 | -0.0205 | 0.9820 |
| NP_001009393 | Antithrombin-III precursor^*^ | SERPINC1 | 88 | -0.1598 | 0.0226 | -0.0709 | 0.1266 | -0.0164 | 0.8673 |
| XP_012013061 | Heparin cofactor 2 isoform X2^†^ | SERPIND1 | 66 | -0.0387 | 0.6746 | -0.1590 | 0.0757 | -0.0425 | 0.3756 |
| XP_012040454 | Pigment epithelium-derived factor isoform X1^†^ | SERPINF1 | 84 | -0.1793 | 0.1240 | -0.2382 | 0.0008 | -0.0967 | 0.1876 |
| XP_012006487 | Alpha-2-antiplasmin isoform X2^†^ | SERPINF2 | 68 | -0.0975 | 0.5498 | -0.1381 | 0.2211 | -0.0725 | 0.6039 |
| XP_012008012 | Plasma protease C1 inhibitor isoform X3^*, †^ | SERPING1 | 68 | 0.0899 | 0.0468 | 0.0112 | 0.6630 | 0.0578 | 0.3242 |
| XP_011977976 | Sex hormone-binding globulin isoform X1^†^ | SHBG | 77 | 0.1303 | 0.3690 | -0.1710 | 0.4503 | 0.0432 | 0.8099 |
| XP_012027928 | Secreted phosphoprotein 24 isoform X1^†^ | SPP2 | 64 | 0.1087 | 0.5999 | 0.0439 | 0.8038 | -0.5105 | 0.3377 |
| XP_011958592 | Serotransferrin isoform X1 (TF) ^*, †^ | TF | 69 | 0.0924 | 0.6227 | -0.1529 | 0.0350 | 0.0922 | 0.1706 |
| XP_011958617 | Inhibitor of carbonic anhydrase-like isoform X1^†^ | TF | 65 | 0.2035 | 0.6196 | -0.2107 | 0.3297 | 0.2826 | 0.7383 |
| XP_012026077 | Inhibitor of carbonic anhydrase-like isoform X4^†^ | TF | 65 | -0.0383 | 0.9182 | -0.0769 | 0.5183 | -0.0015 | 0.8390 |
| XP_012026079 | Serotransferrin isoform X2^†^ | TF | 69 | -0.0705 | 0.4799 | 0.1438 | 0.5554 | 0.0594 | 0.5681 |
| XP_011980737 | Transferrin receptor protein 1^†^ | TFRC | 76 | -0.0968 | 0.5984 | -0.1156 | 0.0729 | 0.0210 | 0.8232 |
| XP_012011958 | Tenascin isoform X18^*, †^ | TNC | 87 | 0.0534 | 0.4515 | -0.2142 | 0.0484 | -0.8584 | 0.0141 |
| XP_012014161 | Pantetheinase isoform X3^†^ | VNN1 | 83 | 0.0138 | 0.7467 | -0.1467 | 0.0270 | 0.0024 | 0.7978 |
| XP_011998146 | Vitronectin isoform X2^†^ | VTN | 74 | -0.0861 | 0.2713 | -0.1567 | 0.0547 | -0.1890 | 0.0605 |

**Supplementary Table 3: Summary of the genes investigated in this study.** The corresponding probe from the Universal Probe Library is also specified (UPL#).

| **Gene** | **Accession Number** | **Primer Sequence (5’-3’)** | **Universal Probe Library Number** |
| --- | --- | --- | --- |
| IL1B | NM_001009465 | F-gcagtgcggtcatcgtg  R-catcacggaagacatgttcg | 44 |
| IL6 | NM_001009392 | F-tgaaggaaaagatcgcaggt  R-cttctccagcatgtcagtgtg | 19 |
| IL8 | NM_001009401 | F-agctggctgttgctctcttg  R-cagaactgcagcttcacacag | 15 |
| CTGF | NM_001164714.1 | F-gacccccaagatctccaag  R-atggctcgatttaagacgcc | 23 |
| CYR61 | XM_004003573 | F-aacatcagtgcacatgtatcgac  R-tggggagagagagttcttgg | 6 |
| EGR1 | NM_001142506 | F-cagcagccccatctactcc  R-ggctcagggaagatgtcagt | 60 |
| RPS15 | XM_012121306.1 | F- gggtaagctgtggcctaaaa  R- cggctgcaacgaggtaaa | 12 |
